# Whole-Genome Sequencing of Lysobacter capsici VKM B-2533^T^ and Lysobacter gummosus 10.1.1, Promising Producers of Lytic Agents

**DOI:** 10.1128/mra.00484-22

**Published:** 2022-08-03

**Authors:** Sergey V. Tarlachkov, Irina V. Kudryakova, Alexey S. Afoshin, Elena A. Leontyevskaya, Natalia V. Leontyevskaya (Vasilyeva)

**Affiliations:** a G.K. Skryabin Institute of Biochemistry and Physiology of Microorganisms, FRC PSCBR, Russian Academy of Sciences, Pushchino, Moscow Region, Russia; Indiana University, Bloomington

## Abstract

Lysobacter capsici VKM B-2533^T^ and Lysobacter gummosus 10.1.1 are promising strains for use in biomedicine as sources of new antimicrobial agents. Here, we report the whole-genome sequences of both strains (total lengths, 6,239,188 bp and 6,056,609 bp, respectively), obtained using the Illumina and Nanopore platforms.

## ANNOUNCEMENT

Some bacteria of the genus *Lysobacter* are capable of producing various antimicrobial agents, such as antibiotics, bacteriolytic enzymes, and peptides (1–7). Despite their great promise for biomedicine, these bacteria have been studied very poorly. It is imperative to search for, isolate, and characterize new lytic agents, as well as to conduct genetic studies of *Lysobacter* strains active against living pathogenic bacteria.

Lysobacter capsici VKM B-2533^T^ (=KCTC 22007^T^ = DSM 19286^T^) is a promising strain with antimicrobial activity (8). It was isolated in 2008 from the rhizosphere of peppers at Gyeongsang National University (Jinju, South Korea) (9). Previously, we conducted draft genome sequencing of this strain (10). Here, we report its whole-genome sequence.

Lysobacter gummosus 10.1.1 is also a promising strain with antimicrobial activity. It was isolated from a disease suppressive soil at Wageningen University and Research (Ijzendijke, Netherlands) (11). It has been shown that *L. gummosus* 10.1.1 has protease, glucanase, and chitinase activities, as well as antifungal and antibacterial activities against Xanthomonas campestris (12).

*L. capsici* VKM B-2533^T^ was obtained from the All-Russian Collection of Microorganisms (VKM). *L. gummosus* 10.1.1 was supplied by Joeke Postma (Wageningen University and Research, Netherlands). Both strains were cultivated in a modified LB medium (13) at 29°C with aeration for 18 h. DNA was extracted using the Wizard genomic DNA purification kit (catalog number A1125) according to the manufacturer’s instructions.

Sequencing on the MinION platform was performed by the Institute of Cell Biophysics, Russian Academy of Sciences (Pushchino, Russia). Libraries for sequencing were prepared using the ligation sequencing kit (SQK-LSK109; Oxford Nanopore Technologies) and native barcoding expansion 13-24 kit (EXP-NBD114; Oxford Nanopore Technologies) according to the manufacturer’s protocol. The resulting libraries were loaded onto a MinION flow cell R9.4.1 (FLO-MIN106; Oxford Nanopore Technologies) and sequenced using MinKNOW v21.10.4 for 16 h.

Short-read sequencing for *L. gummosus* 10.1.1 was performed by the Genomics Core Facility at the Skolkovo Institute of Science and Technology. The library was prepared with the NEBNext Ultra II kit (New England Biolabs, USA) according to the manufacturer’s recommendations. The library was sequenced on the HiSeq 4000 platform (Illumina, USA) to obtain 151-bp paired-end reads. Short-read data for *L. capsici* VKM B-2533^T^ was reused from our previous work (10).

Default options and recommended procedures were used for all software unless otherwise noted. Long-read base calling was performed using Guppy v4.5.4 (Oxford Nanopore Technologies) with the options “-c dna_r9.4.1_450bps_hac.cfg –barcode_kits EXP-NBD114 –trim_barcodes.” Short and low-quality reads were removed using Filtlong v0.2.1 (https://github.com/rrwick/Filtlong) with the options “–min_length 3000 –keep_percent 90.” The genome backbone was assembled using Canu v2.2 (14) with the options “genomeSize = 6050k -nanopore” (for *L. gummosus*) or “genomeSize = 6270k -nanopore” (for *L. capsici*) and then manually circularized. The assembly was polished with the long reads using Nanopolish v0.13.3 (15) with the option “–fix-homopolymers.” Further polishing was carried out using the short reads. Adapter sequences and low-quality regions in the short reads were removed using Trimmomatic v0.39 (16) with the options “ILLUMINACLIP:TruSeq3-PE-2.fa:2:30:10 SLIDINGWINDOW:4:20 MINLEN:50.” The assembly was polished using Polypolish v0.5.0 (17) and Pilon v1.24 (18) with the options “–fix bases –mindepth 5.” The annotation was performed using the NCBI PGAP v6.0 (19).

Statistical information for the whole-genome sequences of *L. capsici* VKM B-2533^T^ and *L. gummosus* 10.1.1 is given in Table 1. The whole-genome sequence of the *Lysobacter* strains obtained in this work will make it possible to find the genes of new lytic agents and to study them. All this will enable the creation of antimicrobial drugs against superbugs in the future.

**TABLE 1:** Statistical information for genome sequences and DDBJ/ENA/GenBank accession numbers

Characteristic	Data for strains	
	*L. capsici* VKM B-2533^T^	*L. gummosus* 10.1.1
No. of long reads	28,810	29,063
Long-read *N*_50_ (bp)	32,612	30,409
No. of short reads	13,667,678	24,335,018
Coverage (×)	206	434
Genome size (bp)	6,239,188	6,056,609
G+C content (%)	66.9	66.5
No. of protein-coding genes	5,030	4,894
No. of hypothetical proteins	1,195	1,002
No. of tRNAs	53	51
No. of rRNAs	6	6
No. of ncRNAs^*a*^	4	4
SRA accession no.		
Long reads	SRR18590105	SRR18590331
Short reads	SRR12790239	SRR18590309
GenBank accession no.	CP094357.1	CP093547.1

a ncRNAs, noncoding RNAs.

### Data availability.

These whole-genome shotgun projects have been deposited at DDBJ/ENA/GenBank under the accession numbers listed in Table 1. The versions reported here are the first versions.
